# Prospective associations, longitudinal patterns of childhood socioeconomic status, and white matter organization in adulthood

**DOI:** 10.1002/hbm.25031

**Published:** 2020-06-12

**Authors:** Alexander J. Dufford, Gary W. Evans, Julia Dmitrieva, James E. Swain, Israel Liberzon, Pilyoung Kim

**Affiliations:** ^1^ Department of Psychology University of Denver Denver Colorado USA; ^2^ Department of Design and Environmental Analysis and Department of Human Development Cornell University Ithaca New York USA; ^3^ Department of Psychiatry and Behavioral Health, Psychology, and Obstetrics, Gynecology, and Reproductive Health Renaissance School of Medicine at Stony Brook University Stony Brook New York USA; ^4^ Department of Psychiatry Texas A&M University Health Science Center College Station Texas USA

**Keywords:** brain development, childhood poverty, diffusion tensor imaging, family income, longitudinal, white matter

## Abstract

The association between childhood socioeconomic status (SES) and brain development is an emerging area of research. The primary focus to date has been on SES and variations in gray matter structure with much less known about the relation between childhood SES and white matter structure. Using a longitudinal study of SES, with measures of income‐to‐needs ratio (INR) at age 9, 13, 17, and 24, we examined the prospective relationship between childhood SES (age 9 INR) and white matter organization in adulthood using diffusion tensor imaging. We also examined how changes in INR from childhood through young adulthood are associated with white matter organization in adult using a latent growth mixture model. Using tract‐based spatial statistics (TBSS) we found that there is a significant prospective positive association between childhood INR and white matter organization in the bilateral uncinate fasciculus, bilateral cingulum bundle, bilateral superior longitudinal fasciculus, and corpus callosum (*p* < .05, *FWE* corrected). The probability that an individual was in the high‐increasing INR profile across development compared with the low‐increasing INR profile was positively associated with white matter organization in the bilateral uncinate fasciculus, left cingulum, and bilateral superior longitudinal fasciculus. The results of the current study have potential implications for interventions given that early childhood poverty may have long‐lasting associations with white matter structure. Furthermore, trajectories of socioeconomic status during childhood are important—with individuals that belong to the latent profile that had high increases in INR having greater regional white matter organization in adulthood.

## 1 INTRODUCTION

Childhood socioeconomic status (SES) is associated with neurobiological development such as reductions in measures of brain structure obtained magnetic resonance imaging (Brito & Noble, 2014; Johnson, Riis, & Noble, 2016). Most of the evidence linking SES and brain development has focused on gray matter structure, typically volume or surface measures (Brito & Noble, 2014). Lower childhood SES has been associated with lower hippocampal volumes (Dufford, Bianco, & Kim, 2019; Hanson, Chandra, Wolfe, & Pollak, 2011; Jednoróg et al., 2012; Luby et al., 2013), and the literature has been mixed concerning the direction of the relation between childhood SES and amygdala volume (Dufford et al., 2019; Luby et al., 2013; Merz, Tottenham, & Noble, 2018). However, there is much less evidence of the relations between childhood SES and white matter structure in the brain. We know that a critical aspect of brain development is the gradual process of integration of information across brain regions and that more mature circuitry can communicate across distant brain regions with greater efficiency (Fair et al., 2007; Fair et al., 2009). It is critical to understand the association between childhood SES and white matter development as the maturation of white matter tracts in childhood is association with functioning in a wide variety of processes including cognitive functioning (Nagy, Westerberg, & Klingberg, 2004), emotion regulation (Fields, 2008), and language development (Wong, Chandrasekaran, Garibaldi, & Wong, 2011). Interestingly, these domains are typically found to be associated with childhood SES (Noble, McCandliss, & Farah, 2007; Noble, Norman, & Farah, 2005). Further, the protracted and nonuniform developmental trajectory of white matter structure in the brain may make it particularly “stress‐sensitive” as white matter tracts, such as those connecting the limbic to prefrontal regions, continue to develop into early adulthood (Hanson, Knodt, Brigidi, & Hariri, 2015; Ho et al., 2017). Studies thus far examining childhood SES and white matter structure have been mostly cross‐sectional, therefore it is unclear if (a) childhood SES has a prospective association with white matter structure later in life and (b) how changes in SES across time may be associated with white matter structure.

While there is evidence for certain periods of development being particularly stress sensitive for brain development (Hanson et al., 2015; Ho et al., 2017), the lack of studies of changes in environmental influences across development begs the question if the relation between environmental influences and brain development may also have associations with the amount of change or “dynamics” with the environmental factor. Family income dynamics have been associated with developmental outcomes such that increases in family income are associated with better outcomes and decreases in family income are associated with more developmental difficulties (Dearing, McCartney, & Taylor, 2001, 2006); however, few studies have examined how family income dynamics across development may be associated with brain development. One study examined changes in family income in adolescents (age 10–16) in which family income was measured annually. Using resting state fMRI, Weissman, Conger, Robins, Hastings, and Guyer (2018) found that the mean income over time and the amount of change (the slope), were not associated with variations in resting state functional connectivity between the posterior cingulate cortex (PCC) and the medial prefrontal cortex (mPFC) (Weissman et al., 2018). However, the interaction of mean income and change in income from ages 10–16 (reported yearly) was significant. For families on the lower end of the income distribution, connectivity between the PCC and mPFC depended on their income slope (changes in income from 10 to 16) such that more positive income slopes were associated with greater connectivity with the right inferior frontal gyrus (with the PCC) and right inferior frontal gyrus and left superior parietal lobule (with the mPFC). While the relation between changes in family income across development and resting state functional connectivity has been examined, the relation between changes in childhood SES across development and white matter structure remains unclear. Further the Weissman study was limited to adolescence, therefore it is unclear how changes in SES across larger periods of development (i.e., childhood to adolescence to adulthood) are associated with white matter structure in adulthood.

Using diffusion weighted imaging (DWI), the diffusion of water molecules can be assessed by fitting a tensor model at each voxel using diffusion tensor imaging (DTI) (Le Bihan et al., 2001). For each voxel a measure of restricted diffusion, fractional anisotropy (FA), has been used to study white matter organization in the brain. FA varies from 0 to 1 in which higher values reflect greater anisotropy and therefore greater white matter organization (Alexander, Lee, Lazar, & Field, 2007). DWI has emerged as a useful imaging modality to study the development of white matter structure in the brain (Lebel, Treit, & Beaulieu, 2019; Snook, Paulson, Roy, Phillips, & Beaulieu, 2005). Consistent evidence shows that development from childhood to adulthood is associated with an increase in FA across multiple white matter tracts in the brain (Lebel et al., 2019; Lebel, Walker, Leemans, Phillips, & Beaulieu, 2008). However, the rates of white matter development vary regionally across the brain (Lebel & Beaulieu, 2011). Specifically, frontal‐temporal tracts and their connections have a protracted developmental trajectory with FA increases into early adulthood (Lebel et al., 2019). White matter tracts, such as the uncinate fasciculus and cingulum bundle are known as frontolimbic tracts as they connect the limbic system to the frontal lobes. These frontolimbic tracts have become the focus of several recent studies due to their protracted development as well as their relation to anxiety and depressive symptomology (Olson, Von Der Heide, Alm, & Vyas, 2015; Von Der Heide, Skipper, Klobusicky, & Olson, 2013). In accordance with the uncinate fasciculus and cingulum bundle protracted development, these tracts have been hypothesized to be “stress‐susceptible” (Hanson et al., 2011; Ho et al., 2017; Olson et al., 2015; Von Der Heide et al., 2013). This refers to the tract having a large window of exposure to environmental influences, such as stressful experiences, which may alter the white matter structure through mechanisms such as experience‐dependent plasticity (Fields, 2008; May, 2011).

While childhood SES is associated with high levels of physiological stress (Baum, Garofalo, & Yali, 1999; Evans, 2004; Evans & English, 2002), the specific relationship between SES and white matter structure has only been examined in a few studies. In adults, lower family income was associated with lower FA in specific white matter tracts such as the uncinate fasciculus and superior longitudinal fasciculus (Gianaros, Marsland, Sheu, Erickson, & Verstynen, 2012). In a study of 3–21 year‐old's, lower family income was associated with lower FA in the right parahippocampal cingulum (part of the cingulum bundle) and right superior corticostriate tract (Ursache, Noble, Pediatric Imaging, & Study, 2016). In another study of middle childhood, lower INR was associated with lower FA in the uncinate fasciculus, cingulum bundle, superior longitudinal fasciculus, inferior longitudinal fasciculus, and corticospinal tracts. Therefore, tract beyond the frontolimbic tracts have been found to be associated with childhood SES, such as the association tract, the superior longitudinal fasciculus. Association tracts refer to tracts that connect cortical areas within ipsilateral hemispheres (Mandonnet, Sarubbo, & Petit, 2018). However, the existing studies examining the relationship between childhood SES and white matter structure are cross‐sectional. Therefore, the study designs limit any conclusions about whether childhood SES predicts white matter structure later in life as well as how changes in family income across development may be associated with white matter structure.

Longitudinal models can be used to examine prospective relations that cross‐sectional designs cannot. For childhood SES, prospective relationships have been identified for functional brain activity during an emotion regulation task (Kim et al., 2013), amygdala function (Javanbakht et al., 2015), and brain structural morphometry (Dufford, Evans, Liberzon, Swain & Kim, under review). In these reports, INR at age 9 was associated with brain structure/function in adulthood, while statistically controlling for concurrent, adult INR. Childhood SES and white matter structure may have prospective relations due to the protracted developmental trajectories of white matter maturation. Interestingly, the white matter tracts that do not reach their maturation peak until early adulthood (such as the uncinate fasciculus and cingulum bundle) (Lebel et al., 2008; Lebel & Beaulieu, 2011), are tracts involved in processes that are typically impacted by the experience of childhood SES such as emotion regulation (Zuurbier, Nikolova, Åhs, & Hariri, 2013) and are implicated in disorders that are more common in individuals who have experienced childhood SES such as anxiety (Baur, Hänggi, & Jäncke, 2012) and depression (Huang, Fan, Williamson, & Rao, 2011; Keedwell et al., 2012; Zhang et al., 2012). However, the prospective relation between childhood SES and white matter structure remains unclear.

As INR may change across development, it is critical to examine how groups experiencing similar family income dynamics may be associated with measures of brain development such as white matter organization. Latent growth mixture model (LGMM) is a statistical technique that is well‐suited to examine this question as it attempts to find homogenous subgroups (known as latent profiles) within heterogenous samples when the data are continuous and longitudinal in nature (Gibson, 1959). However, due to the lack of longitudinal studies of the relation between childhood SES and brain development, the relations between longitudinally modeled latent profiles of family income and brain structure remain unclear. As white matter tracts exhibit protracted developmental trajectories with varying developmental peaks for different tracts, specific white matter tracts may vary in SES susceptibility to experience‐based plasticity due to the tract reaching its developmental peak later in life (Hanson et al., 2015; Ho et al., 2017). Therefore, it is critical to examine if different patterns of change in INR over time are associated with white matter organization in adulthood.

Middle childhood is a developmental period of rapid reorganization of the brain (Elton et al., 2014; Ghetti & Bunge, 2012; Qin et al., 2014) and may be period of increased susceptibility to environmental factors such as childhood SES as evidenced by studies finding a positive association between family income and white matter organization in the uncinate fasciculus, cingulum bundle, and superior longitudinal fasciculus (Dufford & Kim, 2017; Ursache et al., 2016). Childhood SES is typically measured using family income, parental education, parental occupation, or a combination of these measures (Brito & Noble, 2014; Johnson et al., 2016). While there is evidence of relation between parental education and brain structure (Brito & Noble, 2014; Johnson et al., 2016), the most commonly found associations are between family income and brain structure (Johnson et al., 2016). Further, the study is focused on examining changes in SES over time. This also makes family income the most appropriate measure of SES as it has more variability over time than parental education or occupation (DiPrete & McManus, 2000). Therefore, the current study focused on family income as an indicator of childhood SES. However, family income as a measure of childhood SES can be problematic if family size is not considered that is, a family income of $20,000 a year for a family of two is quite different from $20,000 a year for a family of six. Therefore, the current study measure family income using INR which is calculated by dividing the family's total income as calculated by the United States Census guidelines by the number of people living in the home.

Based upon previous studies of the relations between childhood SES and white matter structure, we hypothesize that INR (measured at age 9) will have a prospective and positive relationship with white matter structural organization in the uncinate fasciculus, cingulum bundle, and superior longitudinal fasciculus (Dufford & Kim, 2017; Ursache et al., 2016). Further, we hypothesized based upon these studies, that there will be a significant association between concurrent (measured in adulthood at the time of the scan) INR and white matter organization in overlapping but a smaller subset of regions. However, like previous findings (Weissman et al., 2018), we hypothesized that changes in the INR across childhood would also play a role in white matter organization. Specifically, increases in INR over time will be more positively associated with white matter organization in adulthood than other possible patterns such as INR that stays stable or decreases across time.

## 2 MATERIALS AND METHODS

### 2.1 Participants

Participants were a subsample of a large, prospective, longitudinal study of the association between childhood SES and development in rural counties in the Northeastern United States (Evans, 2003). Low‐income families were oversampled. Initial assessments were acquired when participants were 9 years old and participants were followed up at age 13 (Wave 2), at age 17 (Wave 3) and age 24 (Wave 4). Extensive details about the larger longitudinal study have been reported (Evans, 2003; Evans & Kim, 2012). Regarding the number of participants per wave of data collection, Wave 1 had 341 participants, Wave 2 had 226 of the original participants, Wave 3 had 229 of the original participants, and Wave 4 had 245 of the original participants. Previous studies have provided more details about attrition rates and which participants were dropping out of the study (Evans, 2003; Evans & Kim, 2013). Due to IRB restrictions the data is only available upon reasonable request from the authors.

The neuroimaging subsample was recruited after the completion of this larger study and was composed of individuals that had participated in the larger longitudinal study. The neuroimaging subsample (*n* = 53) have 100% overlap with a study of childhood SES and emotion regulation functional activity in adulthood (Evans & Kim, 2013) and a study of the association between childhood cumulative risk exposure and amygdala structure/function in adulthood (Evans et al., 2016) as well as several other neuroimaging studies (Duval et al., 2017; Evans et al., 2016; Javanbakht et al., 2015; Keedwell et al., 2012; Kim, Ho, Evans, Liberzon, & Swain, 2015; Liberzon et al., 2015; Sripada et al., 2014; Sripada, Swain, Evans, Welsh, & Liberzon, 2014). To be eligible for the neuroimaging study, participants had no prior or current treatment for any psychiatric disorders as determined by a clinician‐administered assessment, the Structural Clinical Interview for DSM‐IV. Participants also had no MRI contraindications that would prevent undergoing the MRI procedures.

Of the 54 participants scanned, one participant could not tolerate the scanner. Therefore, there were 53 participants in which diffusion weighted images were collected. Seven participants were removed due to excessive motion during the scanning (see below for the section on DWI quality control). Thus, the sample size for the current analysis was *N* = 43. This neuroimaging subsample and the larger longitudinal sample did not statistically differ based upon childhood SES (income‐to‐needs ratio, INR), age, sex, ethnicity, or adult SES (INR) (*ps* > .05). Tables 1 and 2 show values for INR, age, sex, ethnicity, and adult SES, the values between samples are within similar ranges and have similar means and standard deviations. Regarding family financial support (support from parents), 27.9% of the participants in the subsample were receiving some family financial support in adulthood on a monthly basis. However, the financial support was minimal (mean = 232.08, *SD* = 171.13, range = 35–500). For the neuroimaging subsample, 34 (79.1%) of participants had moved out of their parent's home, the remaining 9 had not.

**TABLE 1:** Demographic characteristics for the neuroimaging sample (*n* = 43). Early adolescence INR and adolescence INR had missing data (*n* = 39 and *n* = 38, respectively)

	*N* (%)	Mean ± *SD*	Range
Childhood INR (age 9)		1.69 ± 1.08	0.16–3.93
Early adolescence INR (age 13)		2.39 ± 1.60	0.22–5.97
Adolescence INR (age 17)		2.54 ± 1.59	0.60–6.88
Concurrent INR (age 24)		3.44 ± 3.57	0.29–20.52
Age at scan in years (wave 4)		23.74 ± 1.39	20–27
Sex (male)	24 (55.8)		
Race/ethnicity (white/caucasian)	39 (90.7)		
Conditional probability of the high‐increasing profile		16.43 ± 0.34	0–100
Conditional probability of the moderate‐Young‐adult‐decreasing profile		11.47 ± 0.24	0–100
Conditional probability of the low‐increasing profile		72.11 ± 0.41	0–100

**TABLE 2:** Demographic characteristics for the full sample (*n* = 341)

	N (%)	Mean ± *SD*	Range
Childhood INR (age 9)		1.67 ± 1.09	0.10–6.21
Early adolescence INR (age 13)		2.29 ± 1.39	0.10–7.46
Adolescence INR (age 17)		2.66 ± 1.64	0.10–9.54
Adult INR (age 24)		2.64 ± 1.96	0.10–10.00
Age at adulthood data collection		23.55 ± 1.59	19.35–27.86
Sex (female)	167(49.0)		
Race/ethnicity (white/caucasian)	326 (95.6)		

As a part of the larger longitudinal study, trained researchers visited the homes of each participant and conducted interviews with the participant's mother. For the home visits at each wave, the researchers measured cumulative risk exposure, demographic information, and measures of mental health. In adulthood, participants underwent a practice session followed by MRI scanning at the University of Michigan's neuroimaging center.

### 2.2 INR

SES was measured using the INR. INR was calculated by diving the total family income, as calculated by the US census guidelines, by the number of people living in the home. INR was measured at age 9 (childhood), age 13 (early adolescence), age 17 (adolescence) and age 24 (adult). For the neuroimaging subsample, INR was measured again at the time of scanning, this is referred to as “concurrent INR” and used in the neuroimaging analysis as it is the most accurate measured of adult SES as it was measured closest to the neuroimaging scan. However, since the INR in adulthood that was measured as a part of the larger longitudinal study and the concurrent INR measured for the neuroimaging sample were calculated around the age of 24, they are highly correlated (*r* = .87, *p* < .00001). The average American household has an INR of ~2.0 and an INR below 1.0 is considered below the poverty line. The mean INR for childhood was 1.69 ± 1.08. The mean concurrent INR was 3.44 ± 3.57. Rather than imputing the INR data for the neuroimaging sample, three participants were removed for missing the concurrent INR data. The imputation was not performed for concurrent INR as the sample size is quite small for an accurate imputation (McNeish, 2017). The final sample with usable diffusion weighted data included 43 subjects (average age = 23.74, *SD* = 1.39, range = 20–27) with 55.8% males (see Table 1 for more information about the sample).

### 2.3 INR LGMM

A LGMM was conducted using the full longitudinal sample (*n* = 341) due to these analyses requiring sample sizes of at least 250 to have the power to detect an accurate profile classifications; therefore conducting the LGMM on the neuroimaging subsample would be underpowered and unstable (Berlin, Williams, & Parra, 2014; Evans, 2003; Tein, Coxe, & Cham, 2013). A LGMM is like a latent profile analysis (LPA), however it allows within‐profile variation due to continuous growth factors, whereas LPA assumes uncorrelated variables in profiles (which would not be the case for INR). The full sample was like the neuroimaging subsample in the distributions of INR (see Tables 2 and 3) and participants in the neuroimaging sample were chosen to be a representative sample of the full sample. The multiple imputation (MI) procedure in SPSS (Spss, 2011) was conducted on the full sample for missing data. The MI procedure uses an MCMC (Markov chain Monte Carlo) algorithm fully conditional specification (FCS) (Enders, Keller, & Levy, 2018). For this method a univariate model is fit using all the other variables as predictors and imputes missing values for each variable being fit. This continues for the number of iterations specified, which for this imputation was the default value of 10. The LGMM was conducted using childhood INR (age 9), early adolescent INR (age 13), adolescent INR (age 17) and adult INR (age 24) and concurrent INR was not included in the model (concurrent INR was only collected for the neuroimaging subsample). For the imputation procedure, 0 participants had missing data for childhood INR, 115 had missing data for early adolescent INR, 112 had missing data for adolescent INR, and 96 had missing data for adult INR. Therefore, the percentage of missing data for each timepoint is 0, 33.7, 32.8, and 28.1%. For 30% missing data, at a same size of greater than 250, multiple imputation with FCS regression show only slightly inflated Type‐1 error rates for a small effect size (McNeish, 2017). Imputed values were used for these missing data for the LGMM. The LGMM with growth mixture modeling was conducted in MPlus (L. Muthén & Muthén, 2016). Change in INR across the timepoints was modeled with a slope, intercept, and quadratic term and adjusted for time in years between each timepoint of data collection. We tested 2, 3, and 4 profile models and used several model fit indices to determine the most optimal solution.

**TABLE 3:** Latent growth mixture model fits for the full sample (*n* = 341)

Profile #	Loglikelihood H_0_	AIC	BIC	LMR ratio (*p*)	Class size	BLRT (*p*)	Entropy
Profiles = 2	−2,158.000	4,344.000	4,397.647	104.536 (.0009)	61/280	−2,212.509 (.000)	0.862
Profiles = 3	−2,142.740	4,321.480	4,390.454	134.911 (.000)	36/33/272	−2,212.509 (.000)	0.872
Profiles = 4	−2,137.760	4,319.521	4,403.822	147.391 (.3330)	66/12/11/252	−2,212.509 (.000)	0.855

Abbreviations: AIC, akaike information criterion; BIC, bayesian information criterion; LMR, Lo–Mendell–Rubin likelihood ratio; BLRT, bootstrap likelihood ratio test.

### 2.4 Diffusion‐weighted image acquisition

Diffusion‐weighted images were acquired on a 3.0‐T Philips scanner (VA fMRI Center, Ann Arbor, MI). Image acquisition parameters were as follows: TR = 9,000 ms, TE = 83.5 ms, FOV = 220, matrix = 96 × 96, slice thickness = 2.29 mm^3^, directions = 32, 65 sagittal slices of the whole brain. An eight‐channel SENSE head coil was used for acquisition.

### 2.5 Diffusion‐weighted image quality control

Diffusion‐weighted image quality control was conducted using DTIPrep (Oguz et al., 2014). DTIPrep provides both visual and automatic quality control of diffusion‐weighted images including image information and diffusion checks, Rician noise removal, inter‐slice brightness artifact detection including removal of “venetian blind” artifacts and motion within single DWI volumes, coregistration to an iterative average of all baseline images, eddy‐current and motion artifact correct, residual motion detection, and directional artifact detection/correction. Raw DWI images were converted from Dicom to Nrrd format and the default DTIPrep protocol was run for each acquisition. DWI acquisitions that were determined to “fail” the default DTIPrep protocol were removed from the analysis (*n* = 7). DWI acquisition that “passed” the DTIPrep default protocol were visually inspected for any remaining artifact; no images were excluded based upon the visual inspection.

### 2.6 Tract‐based spatial statistics

A diffusion tensor model was fit to each voxel of the DWI data that passed the image quality control procedures using FSL's DTIFIT (Jenkinson, Beckmann, Behrens, Woolrich, & Smith, 2012; Smith et al., 2004). Fractional anisotropy (FA) images for each participant were visually inspected for issues of the diffusion tensor model fitting and no images were excluded. Brain masks were created using FSL's bet (Smith, 2000) and visually inspected for accuracy, with no images needing manual editing. The standard TBSS scripts in FSL were used to prepare the FA images for statistical analysis. FA images were nonlinearly registered FMRIB58_FA standard space, a mean FA image is created, and skeletonized to create the mean FA skeleton for the group. The default value of 0.2 was used as a threshold for the FA skeleton. The scripts resulted in a 4D image that contains all the projected skeletonised FA data to be analyzed using a voxel‐wise approach.

### 2.7 Whole‐brain voxel‐wise regression analyses

We conducted separate whole‐brain voxel‐wise regressions to examine both the prospective associations between childhood INR and white matter organization as well as an additional model that examined the differences (based up on the conditional probability of belong to one profile vs. a reference profile) among the latent profiles of INR. The first model consisted of INR at age 9, concurrent INR, as well as age, sex, and ethnicity as covariates. The second model included group membership (coded as conditional probability of each individual to belong to a given latent profile). In these analyses, one of the latent profiles was chosen as a reference group, and the rest of the profile probabilities were entered into a regression model testing the association with adult white matter organization, controlling for age, sex, and ethnicity. Thus, each of the profile probability variables can be interpreted as the effect of being in a given profile versus the reference profile on white matter organization. A third follow‐up model tested a different reference profile to investigate all pairwise differences. As an exploratory analysis we conducted a fourth whole brain voxel‐wise regression that used INR collected at Wave 4 (adulthood) instead of the INR data collected at the time of the scan (concurrent INR) which included childhood INR, age, sex, and ethnicity as covariates. This analysis was conducted because the study had two measurements of INR in adulthood, one as at Wave 4 (age 24) as part of the larger longitudinal study which we based the LGMM, and one that was collected at the time of the scan (concurrent INR). Although these INR values were highly correlated, given that they were both collected around the age of 24, we conducted the exploratory analysis to compare the results of using Wave 4 INR versus concurrent INR as the predictor in the whole brain voxel‐wise regression.

## 3 RESULTS

### 3.1 Descriptive statistics

Childhood INR was associated with the age at the time of the scan (*r* = −.52, *p* < .0003). Childhood INR was not significantly different between male and female participants (*p* > .61). Childhood INR was also not significant different between white and non‐white participants (*p* > .30). The relation between childhood INR and concurrent INR was trending (*r* = .29, *p* > .058). For more information on the sample characteristics see Table 1.

### 3.2 LGMM of INR

**FIGURE 1:**
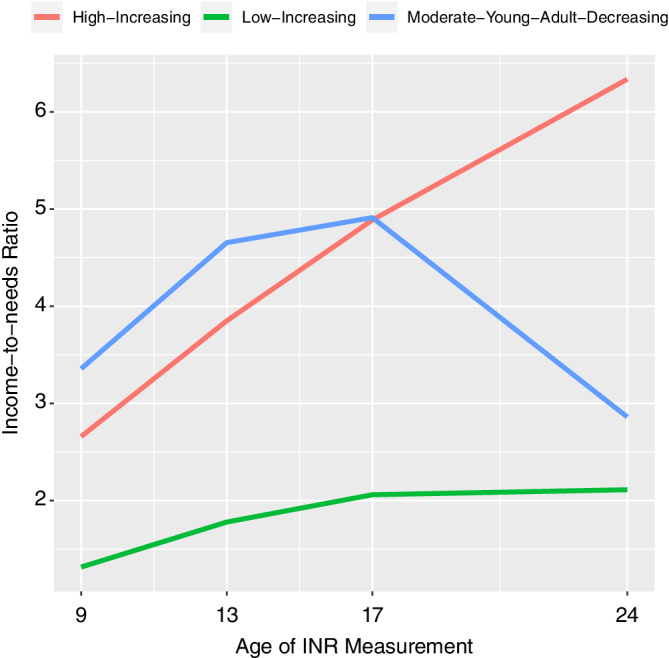
Results of the latent growth mixture model of the full sample (*n* = 341) based upon income‐to‐needs ratio measured at age 9, 13, 17, and 24. The best model fit was for three profiles: one high‐increasing profile (red color), one moderate‐young‐adult‐decreasing profile (blue color), and one low‐increasing profile (green)

Based upon the model fit indices of the 2, 3, and 4 profile solution for the LGMM, the 3‐profile solution was determined to be the best fit (see Table 3 for model fit indices). The BIC and Lo–Mendell–Rubin Likelihood Ratio Test indicated the 3‐profile model as the best fit (BIC decreasing indicates the model fit is improving, increasing means it is not improving; for the Lo–Mendell–Rubin Likelihood Ratio Test, the nonsignificant *p* value indicates the *k* − 1 model or 3 profile), however the parametric Bootstrapped Likelihood Ratio Test *p* value (was *p* < .05) indicated a 4‐profile model. After inspection of the 4‐profile model, there were two profiles that only had 12 and 11 participants assigned to, therefore we determined the 3‐profile model was the best fit model for the data. The three latent profiles determined by the analysis are shown in Figure 1. As hypothesized, the LGMM identified a profile that had high increases in INR from childhood to adulthood. This profile will be referred to as the “high‐increasing profile.” One profile was identified whose INR increased modestly from childhood to adulthood but decreased in adulthood. While unexpected, this decrease is due to individual's family's INR increasing and them becoming financial independent at age 24. This profile will be referred to as the moderate‐young‐adult‐decreasing profile. Lastly, a profile was identified that had low increases in INR and will be referred to as the low‐increasing profile. For the neuroimaging subsample, seven participants were assigned to high‐increasing profile, five participants were assigned to the moderate‐young‐adult‐decreasing profile, and 32 participants were assigned to the low‐increasing profile. For the full sample (*n* = 341), 36 participants were assigned to the high‐increasing profile, 33 participants were assigned to moderate‐young‐adult‐decreasing profile, and 272 participants were assigned to the low‐increasing profile.

### 3.3 Whole‐brain voxel‐wise regression analysis

**FIGURE 2:**
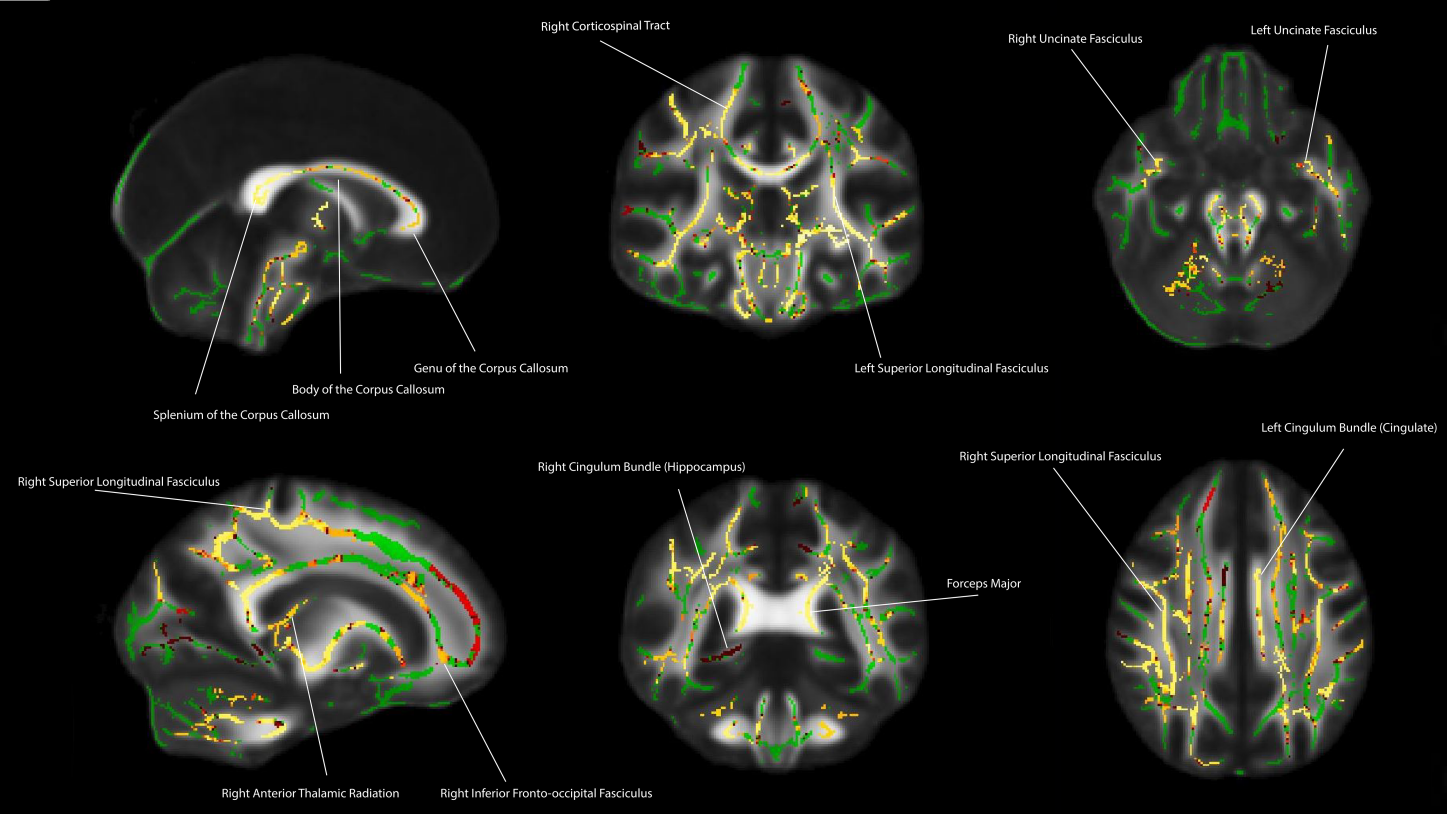
Main effect of childhood income‐to‐needs ratio (at age 9) and white matter organization in adulthood (fractional anisotropy) (*p* < .05, FWE corrected). Covariates included income‐to‐needs ratio at age 24 (concurrent INR), age, sex, and ethnicity. Hot colors reflect regions in which there was a significant prospective and positive association between childhood income‐to‐needs ratio and fractional anisotropy in adulthood. Therefore, lower childhood income‐to‐needs ratio was associated with lower fractional anisotropy in these regions. Hotter colors reflect greater statistical significance for the relation between childhood income‐to‐needs ratio and white matter organization in adulthood

**FIGURE 3:**
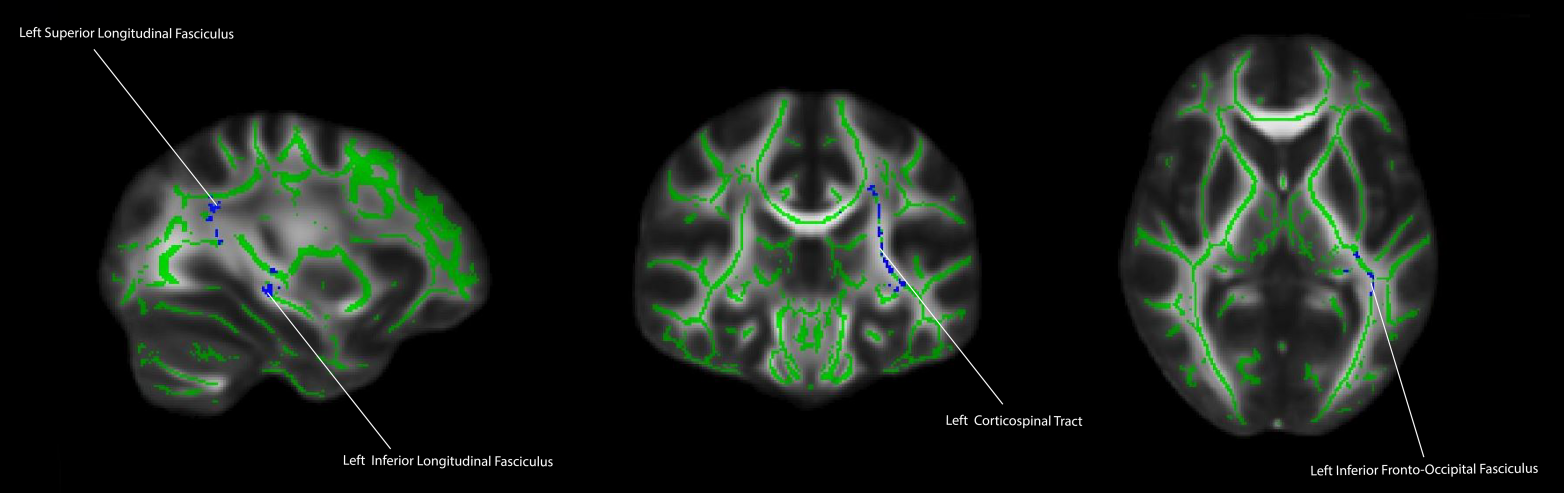
Main effect of adult income‐to‐needs ratio (at age 24) and white matter organization in adulthood (fractional anisotropy) (*p* < .05, FWE corrected). Covariates included age, sex, and ethnicity. Cool colors (blue) reflect regions in which there was a significant positive association between adult income‐to‐needs ratio and fractional anisotropy. Cooler colors reflect greater statistical significant for the relation between adult income‐to‐needs ratio and white matter organization in adulthood

For the first model, we examined the childhood INR contrast to see the main effect of the association between childhood INR and FA. There was a significant positive association between childhood INR and FA in several regions including the bilateral uncinate fasciculus, right cingulum (hippocampus), bilateral cingulum (cingulate gyrus), bilateral superior longitudinal fasciculus, bilateral inferior longitudinal fasciculus, genu, splenium, body of the corpus callosum, bilateral anterior thalamic radiation, bilateral inferior fronto‐occipital fasciculus, forceps major, forceps minor, and bilateral corticospinal tracts (*p* < .05, FWE corrected, see Figure 2). We also examined the contrast for the main effect of concurrent INR, this relationship had a significant positive association at *p* < .05, FWE corrected for the left superior longitudinal fasciculus, left anterior thalamic radiation, left inferior longitudinal fasciculus, left inferior fronto‐occipital fasciculus, and left corticospinal tract (see Figure 3).

**FIGURE 4:**
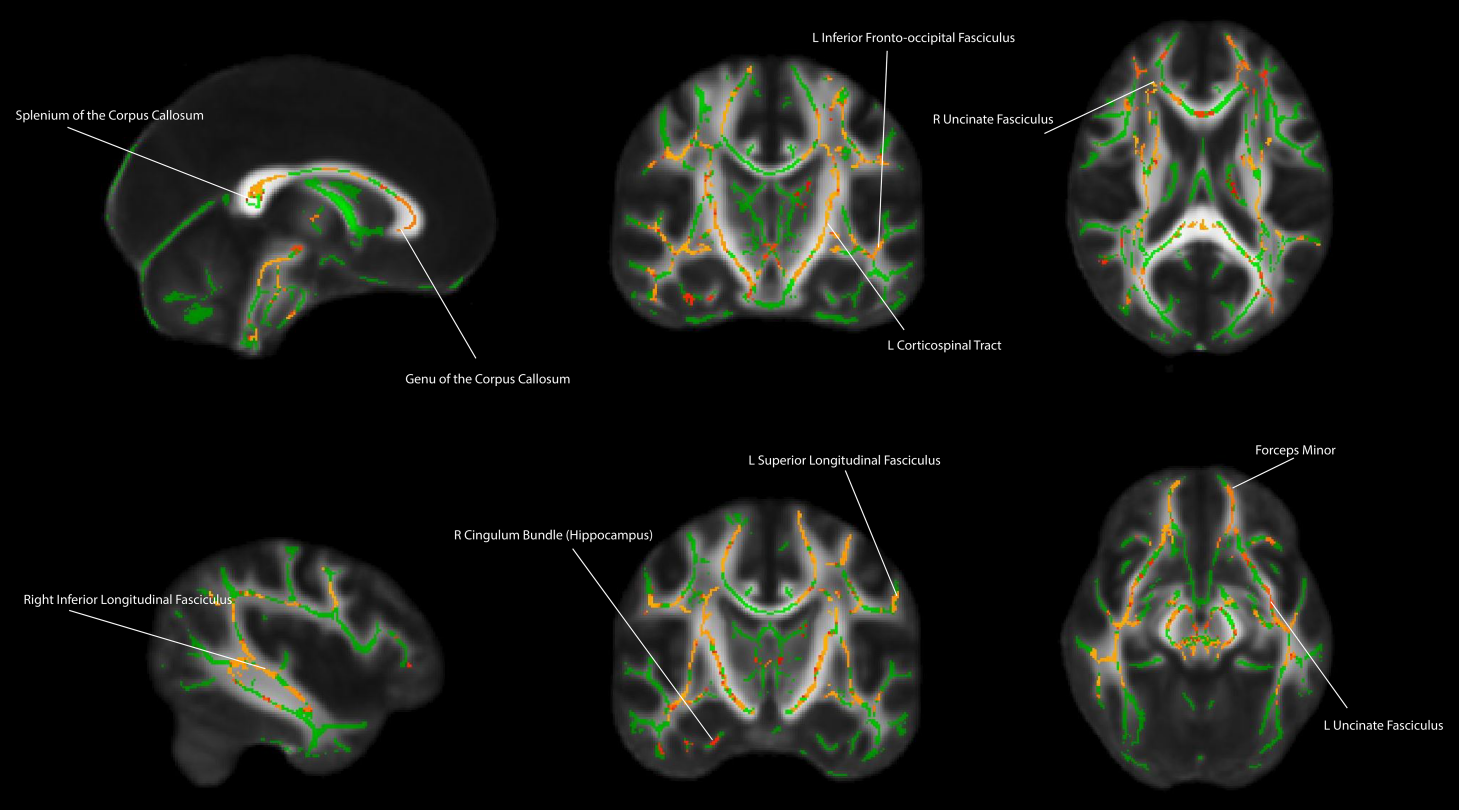
Main effect of the conditional probability that an individual was assigned to high‐increasing profile versus the low‐increasing profile and white matter organization in adulthood. Covariates included age, sex, and ethnicity. Hot colors reflect regions in which there was a significant positive association between conditional probability for membership assignment to the high‐increasing profile versus the low‐increasing profile and fractional anisotropy in adulthood. Hotter colors reflect greater statistical significance for the positive association between the probability of assignment to the high‐increasing profile (vs. the low‐increasing profile) and white matter organization in adulthood

In the second model, we first examined the contrast that tested the association between the conditional probability of being assigned to the high‐increasing profile versus the probability of being assigned to the low‐increasing profile and white matter organization in adulthood. Using conditional probabilities, rather than testing for differences across profiles is advantageous as it considers and quantifies the uncertainty of each individual beings assigned to profile (Muthén et al., 2002; Muthén & Muthén, 2000), whereas testing among groups ignores this uncertainty. Table 1 includes the mean and standard deviations of the conditional probabilities from the LGMM for the neuroimaging subsample. We found a positive association between the conditional probability that each individual was a member of the high‐increasing profile versus the low‐increasing profile and white matter organization in the bilateral uncinate fasciculus, bilateral cingulum (cingulate gyrus), bilateral superior longitudinal fasciculus, bilateral inferior longitudinal fasciculus, genu, body, and splenium of the corpus callosum, forceps minor, anterior thalamic radiation, bilateral inferior fronto‐occipital fasciculus, bilateral corticospinal tract (see Figure 4, *p* < .05, FWE corrected). We also examined the contrast that test the positive association between the conditional probability that each participant was a member of the moderate‐young‐adult‐decreasing profile versus the low‐increasing profile. For the conditional probability of membership in this profile (moderate‐young‐adult‐decreasing profile), there was no significant association at *p* < .05, FWE corrected. In a third whole brain model, we entered in the conditional probability for the moderate‐young‐adult‐decreasing profile and low‐increasing profile (with the high‐increasing profile as the reference group). There was no significant association between moderate‐young‐adult‐decreasing profile conditional probability (in comparison to the high‐increasing profile) and white matter organization at *p* < .05, FWE corrected. For the fourth whole brain model, there was a main effect of INR measured in adulthood that was calculated during larger longitudinal studies and INR that was measured at before the INR at the time of the scan (concurrent INR). There was a positive association between Wave 4 INR and FA in the right anterior thalamic radiation (*p* < .05, FWE corrected) (see Figure S1).

## 4 DISCUSSION

Utilizing a unique sample that combined longitudinal data on income and neuroimaging, we found evidence of (a) a positive prospective association between childhood SES and white matter organization in adulthood in primarily frontolimbic and white matter association tracts such as the uncinate fasciculus, cingulum bundle, superior longitudinal fasciculus; (b) a positive association between concurrent INR and white matter organization in adulthood in primarily association white matter tracts; (c) a positive association between the conditional probability that individuals belonged to a high‐increasing profile for INR versus a low‐increasing profile for INR and white matter organization in adulthood in the uncinate fasciculus, cingulum bundle, superior longitudinal fasciculus; and (d) the results across the prospective analysis and LGMM include several tracts such as the uncinate fasciculus, cingulum bundle, and superior longitudinal fasciculus in common.

There is emerging evidence that experiencing low SES in childhood may have long‐lasting associations with brain structure and function into adulthood. Although there is a dearth of data on SES and brain structure throughout human development, the present findings are consistent with these existing, cross‐sectional studies. For example, we found similar findings of a prospective relationship between childhood SES and brain structure as a cross‐sectional studying examining childhood versus adult SES and hippocampal volume (Yu et al., 2018). Yu et al. reported a positive and prospective relationship between childhood SES and hippocampal volume. We found a similar positive and prospective relationship for childhood SES and white matter organization in several regions including tracts connecting to the hippocampus (cingulum bundle hippocampus). These findings align with the studies that have reported prospective relations of childhood SES with brain structure and function (Javanbakht et al., 2015; Kim et al., 2013; Yu et al., 2018). Thus, converging evidence for this relationship with brain functional activity (Kim et al., 2013), brain surface morphometry (Dufford, Evans, Liberzon, Swain & Kim, under review), and white matter organization, are emerging. However, it is yet unclear how exactly these measures relate to one another. Future longitudinal multimodal neuroimaging studies are needed to examine how these measures are interrelated or how variations in structure contribute to the observed variations in function.

Further, there are common regions among the studies that may indicate why certain developmental outcomes are more common for individuals experiencing childhood SES. For example, childhood SES consistently is associated with frontolimbic structure and function in adulthood (Javanbakht et al., 2015; Kim et al., 2013). Childhood SES was prospectively and inversely associated with ventrolateral and dorsolateral prefrontal cortex activity (and a failure to suppress amygdala activation) (Kim et al., 2013), prospectively and inversely associated with prefrontal surface area in adulthood (Dufford, Evans, Liberzon, Swain, & Kim under review), and prospectively and inversely associated with frontolimbic white matter organization in the uncinate fasciculus and cingulum bundle. These findings suggest that the associations between childhood SES and frontolimbic circuitry may be a potential pathway in which childhood SES influences behaviors that rely on frontolimbic circuitry such as emotion regulation and cognitive control. Furthermore, variations in frontolimbic circuitry have been observed in psychiatric disorders like anxiety (Fu, Taber‐Thomas, & Pérez‐Edgar, 2017; Gee et al., 2016) and depression (Cullen et al., 2010), that children who were exposed to low childhood SES, are more likely to be diagnosed with.

Concurrent INR was associated with white matter organization in portions of the left superior longitudinal fasciculus, left anterior thalamic radiation, left inferior longitudinal fasciculus, left inferior fronto‐occipital fasciculus, and left corticospinal tract. It is worth noting that concurrent INR was not associated with any frontolimbic tracts, but only with association tracts. These findings are supported by studies suggesting that frontolimbic tracts are “stress‐susceptible” due to their protracted developmental trajectories (Hanson et al., 2015; Ho et al., 2017; Lebel & Beaulieu, 2011). While the frontolimbic tracts are reaching their maturational peak around the time that the participants in the current study were scanned, these tracts were undergoing more profound developmental changes in childhood and adolescence than the association tract (Lebel & Beaulieu, 2011; Lebel et al., 2019). This could be one potential explanation for the robust association between childhood INR and the frontolimbic white matter organization in adulthood, while concurrent INR was associated with association tracts in adulthood (see Figure S1a for the concurrent INR results overlaid upon the prospective INR results). However, further investigation of these relations is needed.

As this sample was part of a larger longitudinal study, we utilized the larger sample to extract latent profiles of family income change across development. While LGMM has been used in other tract‐based spatial statistics studies (Rossi et al., 2018; Salami et al., 2018), to our knowledge it has not been used to study the relation between SES and brain structure. Neuroimaging studies of childhood SES are typically cross‐sectional and rarely have two measurements of SES across development to examine changes in SES. The current study had four measurements of SES in childhood, early adolescence, adolescence, and adulthood. The study design provided a unique opportunity to examine white matter organization in adulthood may differ among latent profiles of INR changes across development. Several regions that were observed to have a prospective association with childhood INR, were also found to have associations with the conditional probability that individuals were members of the high‐increasing profile versus the low increasing profile. However, the associations were not observed when comparing the high‐increasing profile versus the moderate‐young‐adult‐decreasing profile and the moderate‐young‐adult‐decreasing profile versus the low‐increasing profile. The moderate‐young‐adult‐decreasing profile has an interesting pattern in which it has higher INR than the high‐increasing profile for the first three time points but then has lower INR than the high‐increasing profile in adulthood. However, the null findings for the high‐increasing profile versus moderate‐young‐adult‐decreasing profile may be due to these values following similar patterns until the measurement in adulthood. The null findings for the moderate‐young‐adult‐decreasing profile versus low‐increasing profile interesting in that the moderate‐young‐adult‐decreasing profile is consistently higher across the first three timepoints and then the profiles converge around an INR of 2.5 and therefore do not have substantial differences in adulthood. In sum, these findings suggest a complex association between changes in INR and white matter organization and demonstrate the substantial divergence in INR in childhood and adulthood may play a critical role in white matter organization in adulthood. These findings also suggest that income dynamics are a critical consideration in future studies childhood SES and brain structure.

While the findings based upon INR changes across development are promising, there are certain caveats worth mentioning. First, LGMM is a data‐driven technique and therefore requires replication in independent samples. Second, the findings should be interpreted with caution because individuals in the latent profile that had the highest increases in INR also typically started out at a high INR values in childhood; however, this concern is mitigated by the high‐increasing profile having a lower starting INR value than the moderate‐young‐adult‐decreasing profile. Due to these considerations, as well as the high collinearity of the INR data across timepoints, we urge caution in strong interpretations regarding developmental timing. These findings need replication in a sample that has individuals that had INR at the low end of the distribution and experienced increases across time to the high end of the distribution. These patterns were not observed in the current sample; therefore, we have made limited conclusions regarding income dynamics and developmental timing.

White matter organization was sensitive to group differences in income dynamics in regions of the uncinate fasciculus, cingulum bundle, and superior longitudinal fasciculus. These regions were common among the analyses. The uncinate fasciculus was significant in all the analyses and its white matter organization has previously been associated with childhood SES in a different, cross‐sectional sample (Dufford & Kim, 2017). The uncinate fasciculus connects the lateral orbitofrontal cortex to the anterior temporal lobes (Von Der Heide et al., 2013). Its developmental peak in early adulthood (also the age the participants in the current study were scanned) has been hypothesized to render it more sensitive to environmental influences for psychiatric illness (Paus, Keshavan, & Giedd, 2008). Due to its connectivity, the uncinate fasciculus is the direct structural link between emotion regulation and higher‐order cognition (Rasmussen et al., 2019). Reductions in white matter organization of the uncinate fasciculus have been observed in several psychiatric disorders including anxiety (Baur et al., 2012), schizophrenia (Kubicki et al., 2002), and conduct disorder (Zhang et al., 2014). Note that each of these disorders is more common in individuals that have experienced low childhood SES (Cohen, 1993; Najman et al., 2010; Wadsworth, Evans, Grant, Carter, & Duffy, 2016).

Portions cingulum bundle was also commonly attuned to income among all the analyses. Previous studies have shown an inverse relationship between childhood SES and white matter organization in the cingulum bundle using cross‐sectional data (Dufford & Kim, 2017; Ursache et al., 2016). The cingulum bundle is a major component of the limbic system, running within the cingulate gyrus, and connects the cingulate cortex to the medial frontal, parietal, occipital, and temporal lobes (Bubb, Metzler‐Baddeley, & Aggleton, 2018). A portion of the cingulum bundle also projects to the hippocampus (Bubb et al., 2018; Goldman‐Rakic, Selemon, & Schwartz, 1984). Reductions in cingulum bundle integrity have been associated with poor emotion regulation ability and implicated in major depressive disorder (Elovainio et al., 2012; Gilman, Kawachi, Fitzmaurice, & Buka, 2002); both of which are more likely in individuals that have experienced childhood SES (Kim et al., 2013; Wadsworth et al., 2016). The superior longitudinal fasciculus was also common among the prospective and LGMM and has been previously linked to childhood SES (Dufford & Kim, 2017). Unlike the uncinate fasciculus and cingulum bundle, the superior longitudinal fasciculus is not directly implicated in affective functioning. However, white matter organization in the superior longitudinal fasciculus has been linked to variations in language ability (Vestergaard et al., 2011) and working memory (Urger et al., 2015); functions that have been demonstrated to be associated with childhood SES (Evans & Fuller‐Rowell, 2013; Evans & Schamberg, 2009; Farah et al., 2006).

Several regions beyond the uncinate fasciculus, cingulum bundle, and superior longitudinal fasciculus were observed across the analyses. Specifically, we did not hypothesize for there to be a prospective association between childhood SES and white matter organization in the corpus callosum, anterior thalamic radiation, inferior longitudinal fasciculus, inferior frontal‐occipital fasciculus, and corticospinal tracts. However, except for the inferior fronto‐occipital fasciculus, these regions have been found to have a positive association with childhood INR in a previous study (Dufford & Kim, 2017). Further studies and replication will be needed to examine the relations between childhood SES, white matter organization, and behavioral outcomes in these tracts. However, like our previous study (Dufford & Kim, 2017), we found that INR is associated with several tracts across the brain that are involved in a multitude of processes including emotion regulation, language, and executive functioning. This is also found in studies of the association between SES and gray matter structure, regions with the association tend to span large portions of the cortex primarily in regions involved in emotion regulation, language, and executive functioning (McDermott et al., 2019; Noble et al., 2015).

The current study should be considered in light of the following limitations. First, while FA is the most commonly examined measure of white matter organization in studies of socioeconomic disadvantage, its interpretability is limited in regions with crossing fibers (Tournier et al., 2008). Future studies will need to examine the relationship between childhood SES and white matter structure using higher‐order tractography such as constrained spherical deconvolution which can avoid the interpretability issues with FA (Tournier, Calamante, & Connelly, 2007). Due to the acquisition parameters of the current study, the higher‐order tractography models could not be examined. Second, childhood SES was first measured at 9 years old, therefore we could not examine SES before this age. Evidence suggests that structural associations with SES may be detectable as early as infancy (Hanson et al., 2013). Third, while we found evidence of a prospective relationship between childhood SES measured at age 9 and white matter organization in adulthood, we cannot make strong conclusions about this developmental period as a “sensitive period” due to the high multicollinearity between the measurements of INR. Fourth, all the profiles experienced some increase in INR across development, therefore future studies will need to examine profiles of families experiencing decreases in INR across development. Fifth, measuring INR at age 24 is difficult and is more heterogenous than the other timepoints of SES measurements as individuals are at various stages of leaving their family to become financially independent. As discussed, this sample had a large percentage of individuals that were financially independent, mitigating this potential confound. Sixth, the sample size was modest and should be replicated in larger samples. Regarding spurious findings, we utilized permutation testing and threshold free cluster enhancement, which for cortical thickness and surface area have shown improved false positive rate control (Greve & Fischl, 2018; Smith & Nichols, 2009). Lastly, the sample was both racially/ethnically homogenous (primarily Caucasian) and did not have past or current diagnoses of mental disorders. Due to the sample not having past or current diagnoses, we did not find it appropriate to examine clinical symptoms in this study. These findings will require replication in samples with more racial/ethnic diversity as well as samples with clinical levels of psychopathology.

## 5 CONCLUSIONS

The current study's longitudinal design afforded the ability to examine critical aspects related to the associations between childhood SES and white matter structure in adulthood that cannot be examine with cross‐sectional studies. Given the prospective association between childhood SES and white matter organization in adulthood, the present findings add weight to the value of efforts to eliminate poverty in childhood. The results from the prospective and concurrent SES analysis suggest childhood SES in middle childhood may play an important role in white matter organization in adulthood. Findings from the LGMM suggest that increases in childhood SES also may play a critical role in the development of white matter structure. In sum, the findings highlight that the association between childhood SES and white matter structure is dynamic and complex and while SES in childhood has a robust association with white matter structure in adulthood; increases in SES across childhood and into adulthood are positively related to the organization of white matter across several white matter tracts in adulthood. The findings may provide support for the policy and interventions to provide income support for families with young children. The support for parents such as job training and education can be effective to increase income for the families for long term, which this pattern has been shown in the current study to be associate with greater white matter organization in adulthood.

## CONFLICT OF INTEREST

The authors declare no conflicts of interest.

## Supporting information


**Figure S1** Supplementary Figure

## Data Availability

Due to IRB restrictions the data is only available upon reasonable request from the authors.
