# Supplementary figures and images for: Prospective associations, longitudinal patterns of childhood socioeconomic status, and white matter organization in adulthood
Source: Hum Brain Mapp. 2020 Jun 12;41(13):3580–93. doi: 10.1002/hbm.25031 (PMC7416042; doi:10.1002/hbm.25031)

a

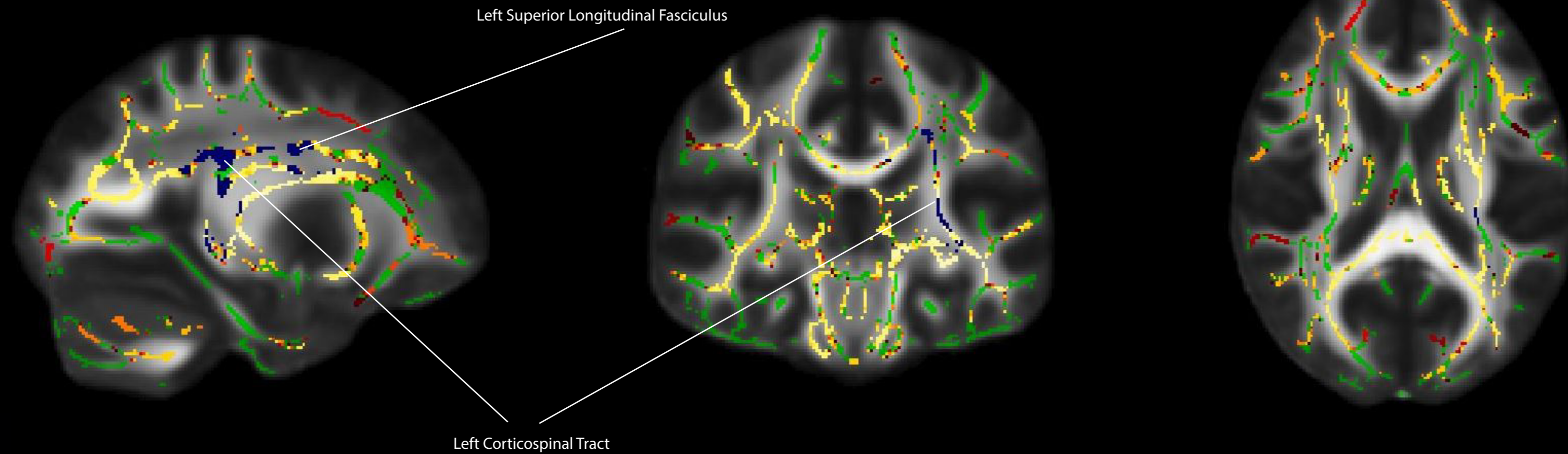

b

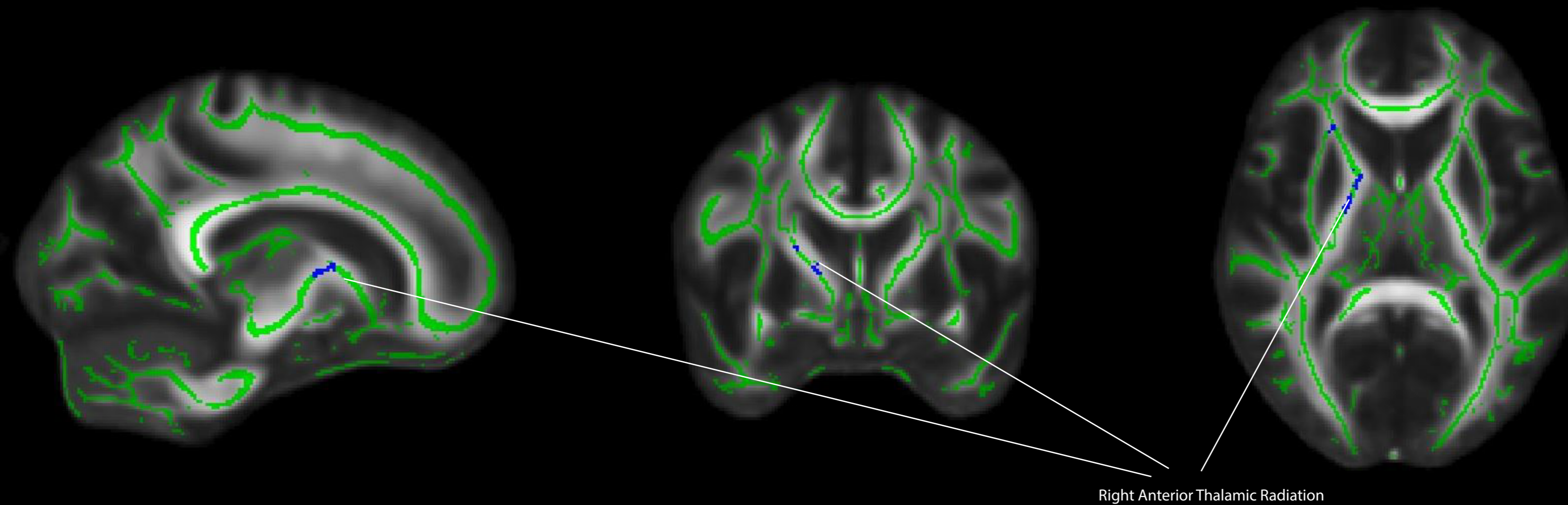

Supplement: Supplementary file 1 — Figure S1 Supplementary Figure [file HBM-41-3580-s001.pdf]
